# A Nationwide Survey of the Quality of Antimalarials in Retail Outlets in Tanzania

**DOI:** 10.1371/journal.pone.0003403

**Published:** 2008-10-15

**Authors:** Harparkash Kaur, Catherine Goodman, Eloise Thompson, Katy-Anne Thompson, Irene Masanja, S. Patrick Kachur, Salim Abdulla

**Affiliations:** 1 Clinical Research Unit, London School of Hygiene & Tropical Medicine, London, United Kingdom; 2 Health Policy Unit, London School of Hygiene & Tropical Medicine, London, United Kingdom; 3 KEMRI/Wellcome Trust Research Programme, Nairobi, Kenya; 4 Immunology Unit, London School of Hygiene & Tropical Medicine, London, United Kingdom; 5 London School of Hygiene & Tropical Medicine, London, United Kingdom; 6 Ifakara Health Research and Development Centre, Dar es Salaam, Tanzania; 7 Malaria Branch, US Centers for Disease Control and Prevention, Atlanta, Georgia, United States of America; Albert Einstein College of Medicine, United States of America

## Abstract

**Introduction:**

Retail pharmaceutical products are commonly used to treat fever and malaria in sub-Saharan African countries. Small scale studies have suggested that poor quality antimalarials are widespread throughout the region, but nationwide data are not available that could lead to generalizable conclusions about the extent to which poor quality drugs are available in African communities. This study aimed to assess the quality of antimalarials available from retail outlets across mainland Tanzania.

**Methods and Findings:**

We systematically purchased samples of oral antimalarial tablets from retail outlets across 21 districts in mainland Tanzania in 2005. A total of 1080 antimalarial formulations were collected including 679 antifol antimalarial samples (394 sulfadoxine/pyrimethamine and 285 sulfamethoxypyrazine/pyrimethamine), 260 amodiaquine samples, 63 quinine samples, and 51 artemisinin derivative samples. A systematic subsample of 304 products was assessed for quality by laboratory based analysis to determine the amount of the active ingredient and dissolution profile by following the published United States Pharmacopoeia (USP) monogram for the particular tablet being tested. Products for which a published analytical monogram did not exist were assessed on amount of active ingredient alone. Overall 38 or 12.2% of the samples were found to be of poor quality. Of the antifolate antimalarial drugs tested 13.4% were found to be of poor quality by dissolution and content analysis using high-performance liquid chromatography (HPLC). Nearly one quarter (23.8%) of quinine tablets did not comply within the tolerance limits of the dissolution and quantification analysis. Quality of amodiaquine drugs was relatively better but still unacceptable as 7.5% did not comply within the tolerance limits of the dissolution analysis. Formulations of the artemisinin derivatives all contained the stated amount of active ingredient when analysed using HPLC alone.

**Conclusions:**

Substandard antimalarial formulations were widely available in Tanzania at the time of this study. No products were detected that did not contain any amount of the stated active ingredient. Quinine and sulfadoxine/pyrimethamine products were the most widely available and also the most likely to be of poor quality. Substandard products were identified in all parts of the country and were labeled as made by both domestic and international manufacturers. With the expansion of the retail pharmaceutical sector as a delivery channel for antimalarial formulations the need for regular nationwide monitoring of their quality will become increasingly important.

## Introduction

*Plasmodium falciparum* malaria is estimated to be the direct cause of 213.5 million clinical episodes per year in Africa and 1.14 million deaths [1]. It is therefore of vital importance that antimalarial drugs administered are genuine and of high quality. Poor quality drugs can be divided into 2 categories: counterfeit and substandard. Counterfeit drugs are deliberately and fraudulently mislabelled with respect to identity, source, or both. Counterfeiting can apply to both branded and generic products and could include products with the correct ingredients or with the wrong ingredients, without active ingredient, with insufficient active ingredient, or with fake packaging [2]. Substandard drugs are genuine drug products that upon laboratory testing do not meet the quality specifications claimed by their manufacturer. This may reflect substandard manufacturing technology, or inappropriate storage and transportation. Many developing countries do not have the technical, financial, or human resources required to inspect and police the drug supply. The World Health Organisation has estimated that about 25% of the medicines consumed in developing countries are counterfeit. In some countries the figure is thought to be as high as 50% [3].

Suspect drugs not only contribute directly to malaria deaths, but may also lead to an increase in the incidence of drug resistance [4], which is among the most important threats to health in tropical countries [5]. Furthermore the presence of counterfeit/substandard drugs in the market undermines public confidence in pharmaceutical products and may result in a reduced uptake of potentially lifesaving medicines [6].

The retail sector represents an important source of antimalarials in Africa; a recent review found that the proportion of caregivers seeking treatment from shops during recent childhood illness ranged from 15% to 83%, with a median across studies of approximately 50% [7]. Little is known about the source and quality of products stocked, although several small-scale studies have documented the presence of poor quality antimalarial drugs in the African retail market [4]. Moreover, with the increase in artemisinin availability and demand, the prevalence of counterfeit products of this new class of drug may spread quickly, following the pattern observed in SE Asia [8].

Existing African studies of antimalarial quality are restricted to relatively small numbers of samples, collected in limited geographical areas, generally using convenience sampling. In this study we undertook the first nationwide study of the quality of antimalarial drugs available in the retail sector in rural Africa. We collected samples of oral antimalarial tablets from retail outlets across mainland Tanzania and assessed them using standard methods for evaluating dissolution and amount of active ingredient. Data collection focused on rural areas, reflecting the geographical pattern of the malaria disease burden in Tanzania. The results document the scale of the problem in Tanzania and will serve as a baseline for the evaluation of trends over time, and the effectiveness of quality improvement strategies. In addition, we investigated risk factors for poor quality (e.g. type of shop, geographical location, generic type, country of manufacture) to help target interventions to improve antimalarial quality.

## Methods

### Background to the Tanzanian retail market for antimalarials

The Tanzanian retail sector plays an important role in antimalarial provision. For example, in 3 rural Tanzanian districts the majority of provider visits for fever/malaria were to retail outlets, which supplied 38% of antimalarial sales volumes [9]. At the time of the collection of these antimalarial samples the first line antimalarial was sulphadoxine-pyrimethamine (SP) or sulphamethoxypyrazine-pyrimethamine (SMP). Amodiaquine was the second line, and quinine the third line but first choice in treatment of severe malaria (SP was replaced as first line by artemether-lumefantrine in late 2006, after this sample collection was complete). Treatment for uncomplicated malaria was provided by hospitals, health centres and dispensaries. However, antimalarials were also widely available from the retail sector, comprising Part I pharmacies, Part II drug stores and general shops. Part I pharmacies were required to be run by a registered pharmacist, and can legally sell any Tanzanian-registered drug, including prescription only and over the counter medicines [10]. These outlets were rare in rural areas. Part II drug stores can be staffed by anyone with a minimum of 4 years medical training (e.g. nurse, pharmacy assistant). They were permitted to stock over the counter products only, such as painkillers and oral formulations of amodiaquine, although it was known that they frequently stocked prescription only products, such as other antimalarials, antibiotics and injectable medications [10]. In 2003 there were 5666 registered Part II drug stores in Tanzania, although in reality this figure is probably much higher [11]. General shops ranged from large shops to small roadside stalls, typically stocking a mixture of food products and household goods, and a few medicines, such as common painkillers and the occasional antimalarial. General retailers were not permitted to sell drugs officially, but in practice, the government allowed the sale of over-the-counter products. These shops were extremely numerous and very accessible, even to rural populations, although only a minority stocked antimalarials [12].

### Sample collection

**Figure 1:**
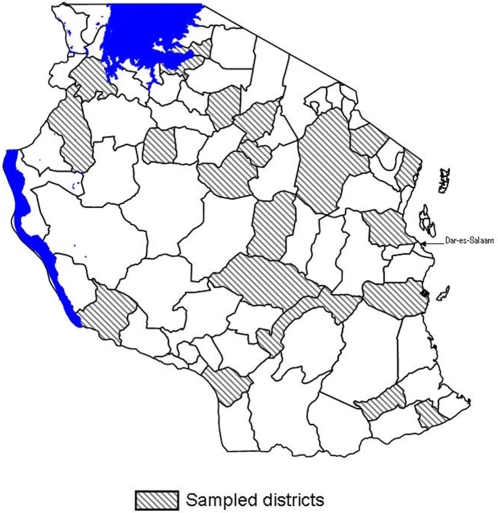
Districts where antimalarial drugs were collection in mainland Tanzania, 2005.

Antimalarial drug samples were collected from 21 of the 121 districts in mainland Tanzania between 16th May and 24th September 2005 (Figure 1). Sample collection was completed as part of a nationwide survey to assess the impact of the Tanzania National Voucher Scheme for insecticide-treated nets and retreatment products [13]. Districts were selected randomly, stratified by the date of implementation of the insecticide-treated net voucher program (early, middle and late implementers). Of the 21 districts, 16 experienced endemic malaria transmission throughout the entire district, while 5 contained epidemic prone areas. In each district 30% of wards ( = n) were surveyed. Each ward was considered to be a “trading centre”. Wards were ordered by the number of businesses registered with the District level Business Office, and divided into major trading centres (MTC) (the 50% of wards with the highest number of businesses) and non-major trading centres (NMTC), the remaining wards. Categorisations were confirmed through discussion with key informants. Half of the sample was made up of the n/2 largest MTC, to reflect the key commercial areas. An additional n/2 centres were randomly selected from the NMTC to represent smaller commercial areas.

In each trading centre all shops (including general shops, drug shops, kiosks, hawkers, pharmacies and supermarkets) were visited and antimalarial tablet samples were collected at each shop where they were stocked on the day of the visit. In total 2474 shops were visited of which 8 were designated as Part I pharmacies, 266 as Part II drugs stores, 2178 as general retailers, and 22 as ‘other’ or ‘unknown’. No drugs were available for purchase in any of the 22 shops designated as ‘other’ or ‘unknown’.

A complete course or adult dose of each of the antimalarial tablet products stocked was purchased for collection from each shop visited. Syrups and injectables were not collected, as they were responsible for a relatively small proportion of malaria treatments obtained from retail sector providers (less than 6% of treatments in one study in 3 rural Tanzanian districts) [9]. A total of 1080 samples were collected. At the time of collection 32 of these products had an expiry date that had already passed and another 51 were set to expire within 6 months of purchase. Data collectors recorded the district, ward and shop where each sample was collected and sent the samples with this information onto the central administrative office of the Ifakara Health Research and Development Centre in Dar-es-Salaam.

The identifying information from each sample was recorded onto an electronic data base, including each product's commercial and generic name, company and point of origin, batch and lot number, and the dates of manufacture and expiration (where available). Samples were retained in their point-of-purchase packaging, sealed in individual plastic bags and stored in cardboard cartons at room temperature until shipping and content analysis. The data base and collected samples were then shipped to the London School of Hygiene and Tropical Medicine for analyses.

### Random tablet selection

To estimate the proportion of substandard products available for a given drug with 10% precision, 95% confidence and estimated proportion substandard = 0.5, we estimated a sample size of 95 for each generic class of antimalarial drug. To arrive at stable estimates of the quality of each generic class collected from the range of outlet types visited, we chose a stratified random sample of products for laboratory analysis. The numbers of antimalarial treatment formulations collected, eligible and selected for content analyses are identified in Tables 1 and 2. Products with no expiry date recorded (n = 32) or that had reached their expiry date prior to analysis (n = 166) were excluded. We analysed a total of 301 of 882 eligible antimalarial tablet products. Of 550 eligible antifolate antimalarial products a sample of 100 was chosen. This included all samples obtained from general retailers and pharmacies (a total of 29). The remaining 71 were chosen randomly from the 521 purchased from drug stores. A total of 58 sulfadoxine/pyrimethamine and 42 sulfamethoxypyrazine/pyrimethamine products were selected. Out of a total of 223 eligible amodiaquine products we also chose a sample of 100. This included all samples collected from pharmacies (total of 7), with the remaining 93 selected from general retailers and drugs stores in equal numbers. All eligible quinine and artemisinin derivative containing tables were analysed, giving a total of 63 quinine and 51 artemisinin derivative -containing monotherapy products respectively. Samples of other antimalarials collected (6 chloroquine, 3 halofantrine and 1 co-packaged mefloquine-artesunate) were not analysed because the sample size was so small. No co-formulated artemisinin-containing combination therapy products were found during data collection.

**Table 1:** Antimalarial drug samples collected and eligible for analyses.

	Collected	Expired before analyses	No expiry data	Eligible for analyses
Amodiaquine	274	36	14	224
Antifol antimalarials	668	107	11	550
Quinine	77	7	7	63
Artemisinin derivatives	51	13	0	38
Chloroquine	6	2	0	4
Halofantrine	3	1	0	2
Mefloquine+Artesunate	1	0	0	1
Total	1080	166	32	882

**Table 2:** Antimalarial drug samples eligible and selected for analyses, by content and source, 2005.

	Antifol antimalarials	Amodiaquine	Quinine	Artemisinin	Total
**Eligible for analyses**					
Part I pharmacy	14	7	2	4	27
Part II drug store	15	139	60	34	248
General shop	521	78	1	0	600
**Total**	**550**	**224**	**63**	**38**	**875**
**Sample selected for analyses**					
Part I pharmacy	14	7	2	4	27
Part II drug store	15	46	60	34	155
General shop	71	47	1	0	119
**Total analysed**	**100**	**100**	**63**	**38**	**301**

Tablet packaging and appearance was assessed, coded, and recorded for each selected product prior to dissolution and content analyses. However, as packaging was not analysed in detail and compared with genuine samples we were unable to distinguish between genuine and counterfeit products. Codes included whether the tablets were sold in blister packages or loose and whether tablets were coated or uncoated. Each selected sample was analysed for quantity of active ingredient using *in vitro* dissolution testing protocols following the detailed monograms outlined in the United States Pharmacopeia (USP) and measuring the amount of active ingredient using high performance liquid chromatographic (HPLC) analyses [14]. The test for content assesses the amount of active ingredient measured in a formulation, expressed as a percentage of the label claim; the test for dissolution determines the amount of active ingredient that is released and available for absorption [4]. Poor manufacturing practices, poor storage of a product as well as the use of incorrect excipients will lead to poor dissolution profiles and thus result in compromised bioavailability. Dissolution testing for pharmaceutical products in tablet and capsule form is required by the US Food and Drug Administration (FDA) and increasingly used outside the USA to report on the quality of drugs.

### Dissolution Analyses

Tablet dissolution was performed in the Pharma Test PT 017 dissolution apparatus (Pharma Test Apparatebau, Hainburg, Germany) using 6 tablets of each product. Dissolution of all antifolate antimalarial products was carried out using 1 litre of 0.01 M pH 6.8 phosphate buffer solution (sodium hydroxide and potassium dihydrogen orthophosphate, Fisher Scientific) and heated to a temperature of 37°C, with a rotor speed of 75 rpm. Dissolution was carried out for 40 minutes and 500 µl samples were taken at ten minute intervals during this time. Of this 500 µl sample 200 µl was transferred into a HPLC reaction vial and diluted 1:1 with 200 µl 0.05 M pH 6.8 phosphate buffer solution and transferred into the HPLC machine for analysis. Dissolution of amodiaquine was performed in 900 mls of purified water and heated to a temperature of 37°C with a rotor speed of 50 rpm for 30 minutes. At ten minute intervals, 500 µl samples were taken and from each of these 200 µl was transferred for HPLC analysis after a 1:1 dilution with purified water. Quinine tablets were subjected to dissolution in 900 mls of 0.1 Molar HCl and heated to a temperature of 37°C, with a rotor speed of 100 rpm. Dissolution was carried out for 1 hour with 500 µl samples taken at ten minute intervals during this time. From each 500 µl sample 200 µl was transferred into a HPLC reaction vial and diluted 1:1 with 200 µl 0.5 M HCl and subsequently transferred into the HPLC machine for analysis.

### Quantity of active ingredient

**Figure 2:**
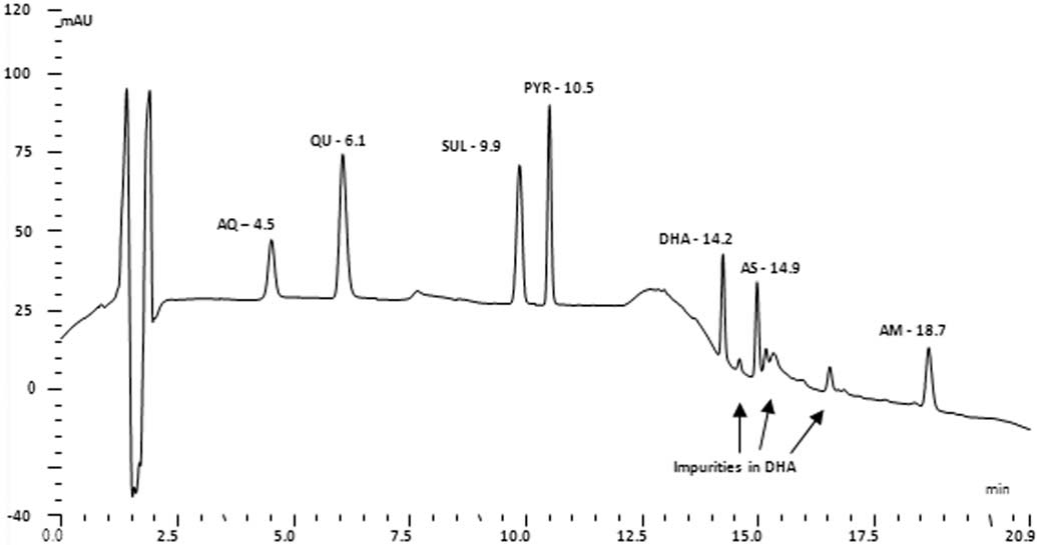
HPLC chromatogram showing the separation of mixture of standards of amodiaquine (AQ), quinine (QU), sulphadoxine (SUL) and pyrimethamine (PYR) all at 10 µg/ml; dihydroartemisinin (DHA), artesunate (AS) and artemether (AM) at 2 mg/ml.

Drug quality was assessed by comparing the amount of active ingredient in the eluents of each dissolution sample against a known concentration of the standard for quinine, amodiaquine, sulfadoxine and pyrimethamine after HPLC analysis (see chromatogram in Figure 2, below for the separation for each compound). Information about the source, packaging or appearance of each product was not known by the investigators prior to analyses of the tablets for quality.

After the dissolution and HPLC analyses the samples were classified as good quality or substandard based on the amount of active ingredient detected listed in Table 3, below. In the absence of a published dissolution monograph for sulfamethoxypyrazine, co-formulations containing this compound were assessed for pyrimethamine content alone. Similarly in the absence of an official monograph for the dissolution profile of artemisinin derivatives the tablets were crushed, dissolved in methanol and an aliquot of the resulting solution was then analysed. The amount of active ingredient detected was determined from a calibration curve plotted using reference standards of each of the artemisinin derivatives (0–10 mgs/ml).

**Table 3:** Classification for content analysis by HPLC for antimalarial drugs.

	Good quality
**Sulfadoxine**	>0.3 mg/ml at 30 minutes
**Pyrimethamine**	>0.015 mg/ml at 30 minutes
**Amodiaquine**	>0.167 mg/ml at 30 minutes
**Quinine**	>0.2025 mg/ml at 45 minutes
**Artemisinin**	>95% of expected concentration

### Data analyses

All data from the initial sample collection data base, packaging and appearance codes and dissolution and chemical content analyses were transferred into STATA version 8 for analysis. Laboratory findings for each generic class of drug obtained from each outlet type were weighted according to the inverse of the probability of selection inherent in the sampling strategy. All estimates and significance tests were corrected for clustering within each shop where products were purchased using the SVY commands. All statistical tests were evaluated at the p< = 0.05 level of precision.

## Results

All of the samples had some active ingredient detected. However, sub-standard products were identified in all generic classes except the artemisinin derivatives (Table 4-note that the calculation of percentages reflects adjustments for different sampling weights across outlet type). Among the antifolate antimalarial tablet products 7 out of 58 (8.6%) sulfadoxine/pyrimethamine tablets did not meet the tolerance limits set by USP for sulfadoxine analysis and one (0.3%) did not meet the tolerance limits set by USP for pyrimethamine analysis. No tablets failed to meet the tolerance limits set by USP for both sulfadoxine and pyrimethamine content analysis. The sulfamethoxypyrazine/pyrimethamine tablets were analysed for pyrimethamine content only. Of these, 9 out of 42 (19.8%) tablets did not meet the tolerance limits set by USP for pyrimethamine and this should be considered conservative since only one of two active ingredients was evaluated. Six of 100 amodiaquine products (7.5%) tested borderline, low or very low. In total 15 out of 63 quinine products (23.8%) did not to meet the tolerance limits set by USP for content analysis, scoring within the borderline, low or very low ranges. All of the 38 formulations of artemisinin derivatives tested were found to contain the expected amount of each active ingredient.

**Table 4:** Numbers and adjusted percentage of samples not meeting the USP tolerance limits for quality test by active ingredient and potential risk factors.

	ANTIFOL∞	SP	SMP∞	AQ	QN	ART	Total
N	100	58	42	100	63	38	301
Total failure	17 (13.4)	8 (8.9)	9 (19.8)	6 (7.5)	15 (23.8)	0 (−)	38 (12.2)
**Outlet Type:**							
General Store	1 (6.7)	0 (−)	1 (33.3)	1 (2.1)	1 (100)	0 (−)	3 (5.3)
Part II Drug Store	10 (14.8)	4 (10.5)	6 (18.2)	5 (10.9)	13 (21.7)	0 (−)	28 (13.5)
Part I Pharmacy	6 (42.9)	4 (50.0)	2 (33.3)	0 (−)	1 (50)	0 (−)	7 (25.9)
P value	0.15	0.07	0.56	0.21	0.13	n/a	0.12
**Packaging:**							
Blister	16 (14.8)	7 (10.3)	9 (20.3)	1 (2.0)	3 (13.6)	0 (−)	20 (11.6)
Loose tablets	1 (1.5)	1 (1.7)	0 (−)	5 (19.2)	12 (29.3)	0 (−)	18 (14.9)
P value	0.007*	0.07	0.62	0.01*	0.17	n/a	0.51
**Appearance:**							
Coated tablet	none	None	None	none	4 (36.4)	0 (−)	4 (36.4)
Uncoated	17 (13.4)	8 (8.9)	9 (19.8)	6 (7.5)	11 (21.2)	0 (−)	34 (11.9)
P value	undefined	undefined	undefined	undefined	0.29	n/a	0.02*
**Stated country of origin:**							
Tanzania	4 (11.9)	3 (11.7)	1 (12.5)	5 (13.2)	7 (18.4)	0 (−)	16 (12.6)
Imported	13 (14.1)	5 (7.3)	8 (21.8)	1 (1.5)	8 (32.0)	0 (−)	22 (12.0)
P value	0.79	0.59	0.58	0.017*	0.22	n/a	0.90
**Size of trading centre:**							
Major trading centre	15 (15.9)	8 (21.9)	7 (10.2)	4 (7.0)	13 (27.1)	0 (−)	32 (12.1)
Non-major trading centre	1 (0.9)	1 (6.0)	0 (−)	1 (8.8)	2 (15.4)	0 (−)	4 (3.9)
P value	0.0002*	0.21	0.22	0.83	0.39	n/a	0.03*

∞ ANTIFOL antimalarials include SP and SMP. SMP samples were tested against only the pyrimethamine standard and should therefore be considered conservative estimates of failure rates.

* Statistically significant at the 0.05 level based on the corrected chi square test.

The proportion of substandard drugs was highest for quinine tablets (23.8%, 95% CI: 14.8, 35.9), followed by antifolate antimalarial tablets (13.4%, 95% CI: 7.7, 22.4) and amodiaquine tablets (7.5%, 95% CI: 3.3, 15.9). The large confidence limits around these estimates reflect the complex sampling strategy and would probably have been smaller if resources had been available to analyse a larger proportion of the samples collected. Details of the chemical content analysis appear in Table 4 below, along with data on the factors associated with poor quality.

There was no statistical association between quality of drug and the type of outlet where it was purchased. Quinine tablets were more likely to be substandard than other antimalarial types, but not significantly so. It is possible that this reflected the high proportion of quinine samples which were obtained loose rather than in blister packs, though again; though the proportion of failures Overall, in bivariate analyses tablets purchased from major trading centres and coated tablets were more likely to be substandard. Since quinine tablets were the only products that were coated, the latter finding may be confounded by the relatively high failure rate of quinine products relative to other antimalarial drug classes. Amodiaquine products sold loose rather than in blister packs were significantly more likely to be sub-standard, but the opposite was found for antifolate tablets, where those in blisters were significantly more likely to fail. Antifolate antimalarial products obtained at major trading centers were substantially more likely to fail quality testing than those purchased in non-major trading centres. This could reflect a concentration of substandard drug in the locations where retail activity is most extensive. While the proportion of imported and locally produced products that failed content analysis was nearly identical, there was a statistically significant association between a stated Tanzanian origin, and poor quality for amodiaquine products. We developed logistic regression models to assess for the multiple predictors of poor quality drug (generic drug class, type of outlet, country of origin, tablet packaging and appearance and size of trading centre), as well as potential interaction terms. None of these factors were found to be independent predictors of substandard medicines (data not shown).

## Discussion

A high prevalence of sub-standard antimalarials in the African retail sector is of great importance in view of the frequency of their use for fever/malaria treatment. Moreover, discussions are currently underway at an international level on strategies to increase access to effective antimalarials through the retail sector through the application of a global subsidy for artemisinin-based combination therapy (ACT). Tanzania has already piloted such a subsidy through Part II drug stores in 2 rural districts [15], and now plans to scale this up nationwide [16].The importance of the retail sector as a delivery channel for antimalarials is therefore likely to increase, further emphasizing the need for regular nationwide monitoring of antimalarial quality.

This study represents the first nationwide survey of antimalarial tablet quality in the African retail sector. Our results showed that poor quality antimalarials were common–12.2% of all samples were substandard, and the figure was as high as 23.8% for quinine, which at the time of the study was the third line antimalarial and first choice for severe disease. The frequency of poor quality formulations was also unacceptably high for antifolate antimalarial drugs (13.4%) which were the first line drug at the time. Moreover, these figures are likely to be an underestimate of true percentages of poor quality as firstly it was not possible to test the dissolution profile for sulphamethoxypyrazine component of the SMPs, and secondly all drugs which had expired by the time of analysis, or had no expiry date recorded were excluded from analysis. No clear predictors of poor quality were identified in this study; substandard antimalarials were obtained from all parts of the country, in major and non-major trading centres, from all types of retail outlet, from both local and international manufacturers, and were identified among both blister packed and loose tablet samples.

Collecting systematic samples of products available in a diverse and far-flung retail market can be a daunting task. In this study, we were able to collect drug samples from major and non-major trading centres in a probability-based selection of districts across mainland Tanzania by joining forces with a separate study on bednets. The method for selection of trading centres within districts was designed to ensure that the most important trading centres were included along with a sample of the smaller commercial centres. However, the sample is not strictly representative in terms of all market centres, and in particular may have under-represented those of medium size. Moreover, we collected one treatment course of all antimalarial tablets that were available on the day of survey, which does not necessarily represent the true range of products that would be obtained and used by consumers. Preferences for particular drug classes, brand names, formulation, packaging and price would all be expected to affect consumer choice. These factors could not be reflected in our sampling strategy. In addition, because they knew they were participating in a research study, shop attendants may have concealed certain unregistered, expired or short-dated pharmaceuticals and products of dubious provenance from data collectors. Even so, our study demonstrates that it is possible to conduct a systematic nationwide assessment of the quality of antimalarial products available in the retail sector and offers a model approach for further research and regulatory work.

We did not assess whether products were counterfeit (i.e. deliberately and fraudulently mislabelled with respect to identity or source) and therefore we only comment on the percent that were found sub-standard. Counterfeit antimalarials have been documented in Africa, for example in Cameroon tablets sold as quinine were found to contain chloroquine instead [17] and counterfeit artemisinin derivatives in Democratic Republic of Congo [Atemnkeng]. The samples were collected from outlets serving the rural population directly. This is important because transport and storage conditions can be a cause of sub-standard medicines [4], a factor which will not be captured when only wholesalers or the main urban pharmacies are sampled. This approach provides a picture of the quality of antimalarials available at the grass-roots; however, it does not allow us to identify the cause of the problem and in particular whether it arose pre or post-factory gate. Little evidence is currently available on antimalarial stability in tropical climates [4].

The frequency of sub-standard antimalarials has varied across studies in Tanzania, but can be difficult to compare across the divergent sampling approaches used. A contemporaneous study from Kilombero and Ulanga Districts of antimalarials obtained from 43 shops and 9 facilities found a higher frequency of sub-standard tablets than our nationwide study, but a similar pattern across generic type: 9% of 35 amodiaquine samples failed on content, compared with 26% of 61 SP samples, and 39% of 33 quinine samples [18]. Two earlier small scale studies in Dar es Salaam also found high frequencies of substandard drugs. Minzi et al's study of 8 wholesalers found 13% of 15 amodiaquine samples failed the dissolution test, but all passed the assay for content, and 11% and 44% of 18 SP samples failed the content and dissolution tests respectively [19]. Risha et al also obtained samples from Dar es Salaam wholesalers, and from the Medical Stores Department in 2000 [20]. Of 4 SP products tested they found that 2 failed the dissolution test. High rates of failure have also been documented elsewhere in Africa. In a recent review Amin and Kokwaro documented 48 studies of content and 30 of dissolution (for multicountry studies each country and each drug class was considered a separate study). In 31 of the 48 content studies more than 80% of samples had appropriate content, although the performance of quinine was noticeably poorer than for other antimalarials. In only 14 of the 30 dissolution studies did more than 80% of samples perform satisfactorily [4].

It was encouraging to note that no artemisinin products failed our quality testing despite a wide range of countries of manufacture including Belgium, France, China, India, Tanzania, and South Korea. However, complacency in this area would be unwarranted. Atemnkeng et al found both sub-standard and counterfeit artemisinin-based products in Kenya and DR Congo [21], and Bate et al found sub-standard products in Ghana, Kenya, Nigeria, Tanzania and Uganda [22]. Moreover, there is strong evidence of high prevalence of counterfeit artemisinin products circulating in South East Asia, usually containing no active ingredient. Surveys in Burma (Myanmar), the Thai/Burmese border, the Lao People's Democratic Republic, Cambodia and Vietnam indicate that 33–53% of artesunate purchased was counterfeit [8]. Artemisinin products are already common in the African commercial sector, particularly in wealthier urban areas. For example, at the time of this study, 19 different artemisinin products were identified from pharmacies in Dar es Salaam [23]. The number is likely to have increased since then, partly in response to the switch to artemisinin-based combination therapy as the first line for treatment of uncomplicated malaria as well as an increasing number of artemisinin-containing products registered with the Tanzania Food and Drugs Authority.

### Conclusion

Antimalarial drugs purchased through the retail sector are one of the key tools used by poor African households to control malaria, and lead to significant household costs. It is therefore essential that the quality and safety of these medicines is assured. This study has demonstrated that a high proportion of retail sector antimalarials in rural Tanzania are of poor quality. However, drug quality is rarely assessed on a large scale, in part due to lack of dedicated laboratory facilities which are expensive to build, equip, set up and maintain in resource poor countries. Similar systematic nation-wide studies of drug quality are warranted on a regular basis throughout the region ensure that the negative consequences of sub-standard drugs are avoided, and that any influx of counterfeit medicines is identified early and addresses.
